# Microvascular dysfunction in patients after successful resuscitation

**DOI:** 10.1186/cc9729

**Published:** 2011-03-11

**Authors:** P Biever, T Schwab, P Roos, O Willnauer, U Denz, K Fink, C Bode, HJ Busch

**Affiliations:** 1 University Hospital Freiburg, Germany

## Introduction

The crucial role of the microcirculation for improved neurological outcome in patients after successful resuscitation has been discussed for many years. Near-infrared spectroscopy has been proposed as a non-invasive tool to measure continuously the haemoglobin saturation in the terminal vascularisation within the tissues (StO_2_) of thenar eminence and to detect microvascular dysfunction by performing a vascular occlusion test (VOT). This study's purpose was to explore the alteration in microcirculation in patients after successful resuscitation.

## Methods

Since August 2010 to date, 23 successfully resuscitated patients were prospectively enrolled in an observational study in the medical intensive care department of Albert Ludwigs University, Freiburg. VOT and the time to recapillarisation were measured at admission to hospital (t1), after induction of mild therapeutic hypothermia (t2) and after re-warming (t3). The VOT was performed by stopping arterial inflow by inflating the arm cuff definitely above the systolic arterial pressure over 3 minutes and recorded with the InSpectra StO_2 _650 monitor (Hutchinson). The recorded StO_2 _alterations were analysed utilising the InSpectra StO_2 _Researcher's Software V 4.01.

## Results

Patients after successful resuscitation showed a baseline StO_2 _of 78.7 ± 8.3%. In all three time points a reduced occlusion slope (t1: -7.2 ± 1.8; t2: -5.8 ± 1.2; t3: 7.6 ± 2.7%/minute) as well as a reduced recovery slope (t1: 1.7 ± 1.1; t2: 1.2 ± 0.7; t3: 1.9 ± 1.7%/second) was seen. Time to recapillarisation was on average 2.7 ± 3.6 seconds.

## Conclusions

Here we could demonstrate important alterations of the tissue-dependent microvascular capacity in patients after successful resuscitation. Considering these data, patients in the post-resuscitation phase may have severe microvascular dysfunction compared with healthy people as described in the literature. This study may highlight a new potentially critical clinical paradigm: extending the duration of mild therapeutic hypothermia may result in favourable neurological outcome by improving post-resuscitation microcirculation.

